# Beyond the Hippocratic Oath: A Planetary Health Pledge for the Malaysian Medical Community

**DOI:** 10.21315/mjms2022.29.1.1

**Published:** 2022-02-23

**Authors:** Jemilah Mahmood

**Affiliations:** Sunway Centre for Planetary Health, Sunway University Malaysia, Selangor, Malaysia

## Introduction

Today, humanity is facing a myriad of crises affecting our collective health as well as that of planet Earth, driven by a complex interplay of political, social, economic, environmental and humanitarian factors. The global SARS-CoV-2 (COVID-19) pandemic has provided unquestionable evidence of the critical relationships between health, environment and economy, and tested the resilience of societies, including in Malaysia where we encountered first-hand the effects of a compounding disaster during the torrential downpours that befell eight states in December 2021 (1), and again, through the unforeseen tornado-like freak storm or fair-weather waterspout in Ipoh (2) just days ago. The World Health Organization estimates that one out of four deaths globally result from exposure to environmental threats (3). As air quality is steadily worsening (4), Malaysians are not exempt from illnesses and deaths related to environmental threats, particularly respiratory and cardiovascular illnesses (5). These crises are glaring reminders of humanity’s long-standing violation of planetary boundaries (6) through indiscriminate and unsustainable development practices that have led to the loss of many lives, disrupted and placed phenomenal burdens on health services, fractured livelihoods and negatively affected mental health.

We now live in the Anthropocene Age, an age where humans are the most significant force shaping the planet’s climate and ecosystems. Without adequate levels of care for the larger planet that we live on, both human health and human development are compromised. Given these current realities, a new approach is needed that emphasises systems leadership, accountability, global and intergenerational equity and solidarity, underwritten by a deep sense of urgency and accepting responsibility for the need to act now.

## Planetary Health: A Vision for the Anthropocene

In response to these challenges, planetary health — a new field and vision — has emerged. The 2015 report (7) of the Rockefeller-Lancet Commission defined planetary health as ‘the achievement of the highest attainable standard of health, well-being and equity worldwide through judicious attention to the human systems — political, economic and social — that shape the future of humanity and the Earth’s natural systems that define the safe environmental limits within which humanity can flourish.’ Over the past century, the focus on public health has improved humanity’s collective wellbeing — but at the expense of the health of the planet. In the 21st century, the human race’s pursuit of its health goals — from improving nutrition to providing healthcare for all — must be balanced with equal concern for the health of the planet. Thus, building healthcare, economic, governance and social systems that are resilient and adaptive to global environmental change need to be prioritised and normalised. A planetary health approach is key to stabilising the global climate, protecting planetary boundaries, and promoting human health and wellbeing in the future, in alignment with the Sustainable Development Goals.

With human health destabilised by increasingly unpredictable environmental changes, current and future generations of health professionals are at the forefront in the fight against the climate crisis. The global health community’s fundamental principle of *primum non nocere* (first do no harm) as enshrined in the Hippocratic Oath naturally translates to not only the great responsibility to care for other people’s lives, but to also do no harm to humanity’s *collective* well-being — including the recognition of the interconnectedness of human health with the state of all natural systems.

## Beyond the Hippocratic Oath

In 2020, concrete language for a Planetary Health Pledge (8) was proposed in *The Lancet*, calling for a unified response and transdisciplinary action to upgrade the Hippocratic Oath to holistically address the diverse challenges that are impeding progress towards the health of both people *and* planet. This means consciously taking action to reduce health impacts of structural inequalities and reflecting the diversity of worldviews and cultural practices, including the respect for indigenous communities’ time-honoured planetary health-oriented views and traditional healing practices. Extending the ‘do no harm’ principle to not only protect human health but also protect the planet is the path that the global health community is now starting to follow and one that the Malaysian health community needs to embrace on a larger scale as a natural step in the Anthropocene epoch.

Initial steps are evident. The term ‘planetary health’ itself is becoming increasingly familiar in Malaysia with grassroots advocates sprouting from the health community, academia and corporate institutions. In 2020, the Malaysian Chapter of the Association of Pacific Rim Universities Global Health Programme released the Kuala Lumpur Statement on Planetary Health (9) urging the academic community to support generating data on planetary health and adopting sustainable individual and institutional practices against environmental degradation and its associated risks to human health. In 2021, the Sunway Centre for Planetary Health was established at the Sunway University (10) with a vision of creating a safe and just world where the health of humans and the planet thrive in harmony, through knowledge, engagement and influence. The Centre has adopted the principle of do no harm as one of its core values, alongside integrity, inclusion, collaboration and stewardship, whilst identifying five priority themes: i) preventing the next pandemic; ii) tackling the climate emergency; iii) creating healthy cities; iv) achieving sustainable food systems and v) promoting fairer economies.

## Conclusion

Safeguarding health for future generations means that the declining state of planet Earth can no longer be ignored. Health professionals, among the most trusted members of society, are well placed to be mediators between science, policy, and practice, and to act as agents for individual and systemic transformative changes towards a healthier and more sustainable humanity.

In the next few editions of this journal, I will dive deeper into some of the more granular elements of planetary health and why it must be a defining priority for all healthcare professionals and educational institutions in this Anthropocene Age. I believe we have an incredible opportunity as health professionals to lead in the transformation of health in Malaysia by:

- being conscious of the need for more sustainable practices in our delivery of healthcare
- advocating the importance of green practices with our patients and partners
- making clear the linkages between emerging diseases and environmental degradation
- reducing our sector’s carbon footprint
- enhancing the resilience of our health infrastructure to withstand the impacts of climate change and
- ensuring sustainable health and food systems, among others

We can do this by taking and fully committing to the Planetary Health Pledge as a first step.
